# The effectiveness of serum S100B, TRAIL, and adropin levels in predicting clinical outcome, final infarct core, and stroke subtypes of acute ischemic stroke patients

**DOI:** 10.7705/biomedica.5943

**Published:** 2022-05-01

**Authors:** Ozge Altintas Kadirhan, Okkes Taha Kucukdagli, Bedia Guien

**Affiliations:** 1 Department of Neurology, Faculty of Medicine, Kirklareli University, Istanbul, Turkey Kirklareli University Kirklareli University Istanbul Turkey; 2 Emergency Clinic, Bakirkoy Dr. Sadi Konuk Education and Training Hospital, Istanbul, Turkey Sadi Konuk Education and Training Hospital Istanbul Turkey; 3 Emergency Department, Faculty of Medicine, Bezmialem Vakif University, Istanbul, Turkey Bezmiâlem Vak?f Üniversitesi Bezmialem Vakif University Istanbul Turkey

**Keywords:** TNF-related apoptosis-inducing ligand, infarction, posterior cerebral artery, stroke., ligando inductor de apoptosis relacionado con TNF, infarto de la arteria cerebral posterior, accidente cerebrovascular.

## Abstract

**Introduction:**

More than half of all worldwide deaths and disabilities were caused by stroke. Large artery atherosclerosis is identified as a high etiological risk factor because it accounts for 20% of ischemic stroke.

**Objectives:**

To identify the significance of TRAIL and adropin release and the relative changes related to S100B levels, as well as the relationship between these biomarkers and the final infarct core, the clinical outcome, and the presence of large artery atherosclerosis in acute stroke patients.

**Materials and methods:**

Over a one-year period, demographic, clinical, and neuroimaging findings of 90 consecutive patients with acute ischemic stroke were evaluated.

**Results:**

The mean age of participants was 69.28 ± 10 and 39 patients were female. The increased level of S100B and the decreased levels of sTRAIL with adropin were significantly associated with moderate to severe neurologic presentation (p=0.0001, p=0.002, p=0.002, respectively). On the control CT, a large infarct core was significantly associated with decreased serum levels of sTRAIL and adropin (p=0.001 and p=0.000, respectively); however, the levels of S100B were not significantly associated with good ASPECTS score (p=0.684). Disability and an unfavorable outcome were significantly related to the decreased level of sTRAIL and adropin (p=0.001 and p=0.000 for THRIVE score>5, respectively). Decreased sTRAIL and adropin levels and an increased S100B level were correlated with the presence of large artery atherosclerotic etiologic factors (p=0.000, p=0.000, p=0.036, respectively).

**Conclusion:**

TRAIL and adropin serum levels were associated with poor clinical outcomes and greater infarcted area in acute ischemic stroke patients.

## Resumen

**Introducción.:**

Más de la mitad de todas las muertes y discapacidades en todo el mundo fueron causadas por accidentes cerebrovasculares. La aterosclerosis de las grandes arterias se identifica como un factor de alto riesgo etiológico debido a que representa el 20 % de los accidentes cerebrovasculares isquémicos.

**Objetivo.:**

Determinar la importancia de la liberación de TRAIL y adropina y los cambios relativos relacionados con los niveles de S100B, así como la relación entre estos biomarcadores y el núcleo final del infarto, el resultado clínico y la presencia de aterosclerosis de arterias grandes en pacientes con accidente cerebrovascular agudo.

**Materiales y métodos.:**

Durante un año, se evaluaron los hallazgos demográficos, clínicos y de neuroimágenes de 90 pacientes con accidente cerebrovascular isquémico agudo.

**Resultados.:**

La edad media de los pacientes fue de 69,28 ± 10 y 39 eran mujeres. El aumento del nivel de S100B y la disminución de los niveles de sTRAIL y adropina se asociaron significativamente con una presentación neurológica moderada a grave en los pacientes (p=0,0001, p=0,002 y p=0,002, respectivamente). En la TC de control, un gran núcleo de infarto se asoció significativamente con una disminución del nivel sérico de sTRAIL y adropina (p=0,001 y p=0,000, respectivamente); sin embargo, los niveles de S100B no se asociaron significativamente con una buena puntuación en el ASPECT (p=0,684). La discapacidad y el resultado desfavorable se relacionaron significativamente con la disminución de los niveles de sTRAIL y adropina (p=0,001 y p=0,000 para una puntuación >5 en el THRIVE, respectivamente). La disminución de los niveles de sTRAIL y adropina y el aumento del nivel de S100B, se correlacionaron con la presencia de un factor etiológico aterosclerótico de arterias grandes entre la población de estudio (p=0,000, p=0,000 y p=0,036, respectivamente).

**Conclusiones.:**

Los niveles séricos de TRAIL y adropina se asociaron con un resultado clínico deficiente y una mayor área infartada en pacientes con ataque cerebrovascular isquémico agudo.

More than half of all deaths and disabilities worldwide are caused by strokes ^1^^,^^2^. Extracranial and intracranial large artery atherosclerosis (LAA) has been identified as a high etiologic risk factor as it accounts for 20% of ischemic strokes ^2^^,^^3^. Diabetes is an independent risk factor for stroke given its involvement in the atherosclerotic process ^4^. Recent research has shown that increased blood glucose levels were associated with large ischemic core volume and poor post-stroke clinical prognosis ^5^.

Dysfunctional endothelium contributes to atherosclerosis initiation and progression ^3^^,^^6^. The tumor necrosis factor (TNF)-related apoptosis-inducing ligand (TRAIL) is part of the TNF superfamily that activates the apoptotic pathways underlying cell damage and disease progression ^7^. Recent studies have shown that TRAIL might contribute to stabilizing atherosclerotic lesions and ameliorating endothelial dysfunction by increasing the release of nitric oxide ^8^. Adropin is a recently identified regulatory protein in the potential biological mechanism of insulin sensitivity and endothelial function ^9^. A previous study in patients with acute myocardial infarction determined that the angiographic severity of coronary atherosclerosis was closely related to low adropin levels ^10^.

S100B is a useful “acute-phase” neurobiochemical marker of brain damage closely associated with plaque instability and oxidative stress in stroke patients ^11^. Based on these findings, we aimed, first, to study the significance of TRAIL and adropin release and the relative changes related to S100B levels in acute stroke patients and, secondly, to analyze the association between these biomarkers and the final infarct core, the clinical outcomes, and the presence of large artery atherosclerosis combined with neuroimaging and clinical risk assessment tools in acute stroke patients.

## Materials and methods

We conducted a prospective observational study at an emergency room (ER) and neurology department over a one-year period. We prospectively studied 90 ischemic stroke patients (ischemic infarct) admitted to the ER in the first 24 h after symptoms onset. Detailed data were registered prospectively for each patient including demographics, vascular risk factors, admission glucose levels, and admission blood pressure levels. A non-contrast computed tomography (CT) brain scan was performed to rule out hemorrhagic stroke on admission. On baseline and the 24-hour control CT, we evaluated the Alberta Stroke Program Early computed tomography Score (ASPECTS). Early ischemic changes in the middle cerebral artery territory were quantified by the ASPECT scoring system where a score of 10 indicates a normal state and one point is subtracted for each infarcted region ^12^. Patients with an ASPECT score of 6 to 10 were classified as having a small infarct core according to previous research ^13^. All patients underwent carotid duplex ultrasonography and magnetic resonance angiography due to diagnosed extracranial and intracranial atherothrombosis.

Large artery atherosclerosis was defined according to the Trial of Org 10172 in Acute Stroke Treatment (TOAST) classification system ^14^. Carotid Doppler ultrasonography and brain imaging methods were used to determine accurately the etiological risk factor. Significant stenosis was defined as a 50% or more diameter reduction in the internal cerebral artery or a narrowing of the vessel lumen due to vascular remodeling following atherosclerosis ^14^.

The National Institutes of Health Stroke Scale (NIHSS) score was calculated for each patient on admission. The modified Rankin Scale (mRS) was recorded on admission and in the first and third months. Patients with mRS 0-2 were classified as having good neurologic outcomes. On admission, we calculated the Total Health Risks in Vascular Events (THRIVE score, including the presence of hypertension, diabetes, and atrial fibrillation, baseline NIHSS score and patient age) for each patient. A THRIVE score from 0 to 5 indicated a good clinical recovery and prognosis ^15^.

After admission to the ER, blood samples were taken from each patient to evaluate the biomarkers under study. The results were recorded in pg/L for sTRAIL and S100B and in ng/ml for adropin after analyzing the samples with the sandwich enzyme immunoassay method according to the manufacturer’s protocol (S100B Elisa Kit: Cat No: E 3039 Bioassay Technology Laboratory Co., Ltd., China; sTRAIL Elisa Kit: Cat No: E 1824 Bioassay Technology Laboratory Co., Ltd., China; Adropin Elisa Kit: Cat No: 201 12 3107 Sunred Technology Laboratory Co., Ltd., China).

All statistical analyses were performed using IBM SPSS Statistics 20 (USA) and the Microsoft Office Excel software. Comparative analyses between the study biomarkers and initial and third-month mRS, initial NIHSS, initial and control ASPECTS, initial THRIVE, and LAA presence were performed using the Chi-square test (x2 test), independent samples t-test, or Mann- Whitney U-test. When the expected frequency was five or less and then five, we used Fischer’s exact test. Age and test values were all expressed as means ± standard deviations (SD). For all tests, a two-tailed P value < 0.05 was considered statistically significant with confidence intervals (Cl) of 95%. A receiver operating characteristic (ROC) curve was constructed to determine the area under the curve (AUC) and the sensitivity and specificity levels of biomarkers for outcome predictions.

### Ethics statement

The Research Ethics Committee at Bezmiâlem Vakif University approved all the study procedures (Decision No: 11/30 June 03/2015).

## Results

Participants’ mean age was 69.28±10 and 39 of them were female (43.3%). The leading vascular risk factor was hypertension (71.1 %). The baseline characteristics of patients are shown in table 1.

**Table 1:** Baseline characteristics of the patients at admission to the emergency room

Characteristics	All study patients (n=90)	(n=90) LAA etiology (+) stroke patients (n=24)	LAA etiology (-) stroke patients (n=66)
Demographics			
Age †	69.28 ± 10	63.88 ± 10,75	71.24 ± 9,02
	(42-87)	(42-77)	(45-87)
Sex			
Female (n, %)	39 (43.3)	12 (13.3)	39 (43.3)
Male (n, %)	51 (56.7)	12 (13.3)	27 (30)
Medical and drug history			
Hypertension (n, %)	64 (71.1)	15 (62,5)	49 (74.2)
Diabetes mellitus (n, %)	12 (13.3)	6(25)	6(50)
Hyperlipidemia (n, %)	27 (30)	8 (33.3)	19 (70.4)
Coronary heart disease (n, %)	20 (22.2)	7 (29.2)	13 (65)
Current smoking habit (n, %)	15(23.3)	1 (4.2)	14 (93.3)

Note: (n, %)

† Mean ± SD (data in parentheses is the range)

LAA: Large artery atherosclerosis; ASPECTS: Alberta Stroke Program Early Computed Tomography Score; NIHSSiThe National Institutes of Health Stroke Scale; THRIVE: Totaled Health Risks in Vascular Events

**Figure 1:**
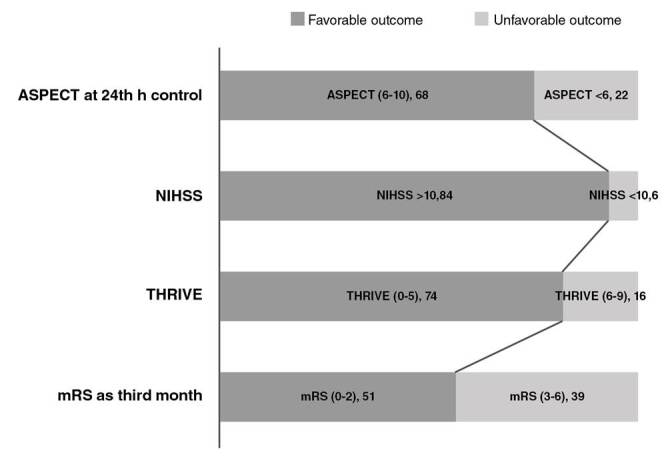
Distribution of baseline and follow-up clinical scores to measure good clinical outcomes and small infarct core in the study patients.

ASPECT: Alberta Stroke Program Early Computed Tomography Score; NIHSS: The National Institutes of Health Stroke Scale; THRIVE: Totaled Health Risks in Vascular Events; mRS: modified Rankin score

Upon admission to the ER, patients had a mean NIHSS score of 17.08±4.65 points; 84 of 90 had moderate to severe stroke with an NIHSS score higher than 10. After the stroke onset, favorable mRS scores of 0-2 were found in 7.8% of the patients (n=7). A significant proportion of patients completely recovered with slight or no long-term disabilities. In the third month, 51 (56.7%) patients had favorable neurologic outcomes with 0-2 mRS scores. Two patients died two months after discharge; 74 (82.2%) had good clinical recovery after the stroke with initial 0 to 5 THRIVE scores. The distribution of baseline and follow-up clinical scores to measure good clinical outcomes and small infarct core are shown in figure 1 .

According to the TOAST stroke subtypes, 24 (26.7%) patients had large artery atherosclerosis, two had internal carotid artery (ICA) occlusion, seven had middle cerebral artery (MCA) M1 segment occlusion, five had unilateral ICA stenosis, and eight had unilateral MCA M1 segment stenosis while only two had bilateral ICA stenosis and no tandem occlusion was observed. In 22 of the patients, we detected a large infarct burden (ASPECTS <6) on control cranial CT scans but no hemorrhagic transformation on the control brain scans.

The increased level of S100B and the decreased levels of sTRAIL and adropin were significantly associated with moderate to severe neurologic presentation corresponding to an NIHSS score higher than 10 (p=.0001, p=.002, p=.002, respectively). On the control CT, a large infarct core was significantly associated with decreased serum levels of sTRAIL and adropin (p=.001 and p=.000, respectively); however, the levels of S100B were not significantly associated with a good ASPECT score (p= .684). Disability and unfavorable outcomes were significantly related to decreased levels of sTRAIL and adropin (p=.001 and p=.000 for THRIVE scores >5, respectively) whereas there was no significant relationship with serum S100B levels (p=.291 for THRIVE score >5). Good prognosis in the third month was also significantly related to sTRAIL and adropin levels (p=.001 and p=.000 for mRS 0-2, respectively) while there was no significant relationship with the level of S100B (p=.291 for mRS 0-2). Decreased levels of sTRAIL and adropin and increased levels of S100B were significantly correlated to the presence of a large artery atherosclerotic etiologic factor (p=.000, p=.000, p=.036, respectively). Clinical and laboratory parameters of the patients at admission to the emergency department are shown in table 2. In a previous study, researchers have shown that adropin is especially related to insulin resistance ^16^. In a subgroup analysis, we found that six stroke patients with LAA had diabetes, a known vascular risk factor, and 12 of them had decreased adropin levels. We did not find any relationship between the study biomarkers and vascular risk factors, admission serum glucose levels, and admission systolic-diastolic blood pressure levels.The biomarkers understudy (sTRAIL, adropin, S100B) were compared with vascular risk factors using the Mann-Whitney test; 39 patients had more than one risk factor (43.3%). The test revealed that only S100B levels were significantly correlated with more than one risk factor (p=.013).

**Table 2:** Clinical and laboratory parameters of the patients at admission to the emergency room.

Characteristics	All study patients (n=90)	LAA etiology (+) stroke patients (n=24)	LAA etiology (-) stroke patients (n=66)	P value
Clinical and laboratory parameters				
Glucose (mg/dl) at admission	138.07 ±63.21	150.9 ±69.9	133.41 ±60.5	.657
	(76-350)	(89-350)	(76-350)	
Systolic blood pressure (mmHg)	154.81 ±33.43	159 ±26	153.3 ±35.8	.233
at admission	(80-240)	(120-230)	(80-240)	
Diastolic blood pressure (mmHg)	80 ± 13.58	82.92 ±12.61	78.95 ± 13.84	.178
at admission	(40-110)	(66-110)	(40-110)	
S100B (ng/L) at admission	976.33 ± 543.75	1174.93 ±611.61)	904.11 ±502.60	.036
	(164.38-2906.44)	(599.62-2678.58)	(164.38-2906.44)	
TRAIL (ng/L) at admission	2542.59 ± 1382.9	3501.82 ± 1353.93	2193.76 ± 1068.42	.000
	(945.01-6580.75)	(1385.11-6580.75)	(945.01-5695.73)	
Adropin (pg/ml) at admission	240.62 ± 91.60	156.75 ±27.1	271.11 ±87.7	.000
	(1131.11 ±455.23)	(113.11 ± 198.42)	(122.62 ±455.23)	
ASPECT score at admission	9.2 ± 1	8.42 ± 1.21	9.48 ± 0.75	.000
	(7-10)	(7-10)	(7-10)	
ASPECT Score at control (at	7.4 ± 1.2	6.2 ± 1.25	7.33 ± 1	.000
24th hr.)	(4-9)	(4-9)	(5-9)	
NIHSS score at admission	17.08 ±4.65	22.38 ± 1.95	15.15 ±3.76	.000
	(4-25)	(19-25)	(4-20)	
THRIVE score at admission	4 ± 1.27	5.2 ± 1.4	3.6 ± 0.92	.000
	(1-7)	(2-5)	(1-7)	

**Figure 2:**
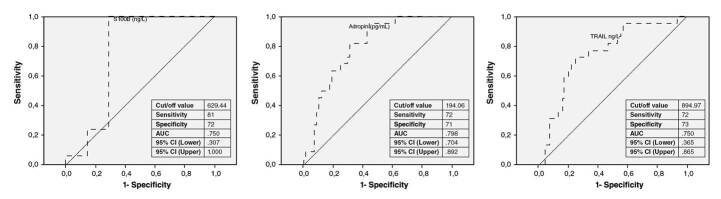
ROC curve analyses of the biomarkers in predicting favorable outcomes in stroke patients

AUC: Area under the curve; ROC: Receiver operating characteristic; Cl: Confidence intervals

We calculated the cut-off values of these biomarkers for our study subjects to determine good clinical outcomes and a small infarct core. For S100B, the cut-off value was 427 ng/L, sensitivity was 100%, specificity was 100%, and the AUC was 1.000 in the ROC curve. For sTRAIL, the cut-off value was 1705.93 ng/L, sensitivity was 86%, specificity was 66%, and the AUC was .143 in the ROC curve. The cut-off value for adropin was 388.7 pg/mL, sensitivity was 98%, specificity was 83%, and the AUC was .028 in the ROC curve. The ROC curve analyses for biomarkers predicting favorable outcomes in stroke patients are shown in figure 2. 

## Discussion

We analyzed 90 acute stroke patients in our study; in 24 of them with large artery atherosclerosis, we found a significant association with the predictor biomarkers for good clinical outcomes and small infarct core.

Ongoing trials have aimed to determine a predictor biomarker to estimate post-stroke survival rate, disability, and final infarct core. S100B is a calcium binding protein that has been a well-known serological biomarker of blood brain barrier dysfunction and damage after cerebral injury ^17^.

Increased levels of serum S100B protein could predict the severity of brain damage and the survival rate. A recent study found that S100B protein was significantly correlated with final brain damage and early neurologic disability in 32 acute stroke patients ^18^. In a study conducted by Weglewski, *et al*., serum S100B protein concentrations in acute ischemic stroke patients admitted to the hospital within 24 hours showed a significant correlation with the final infarct core in those with moderate to severe stroke ^11^. Park, *et al*. examined 111 acute stroke patients and found that serum S100B levels were associated significantly with early and late neurologic disability ^19^. In our study, the evaluation of the biomarkers were measured at a single time within 24 hours after stroke onset. Many studies have showed that the levels of S100B protein could gradually rise within three days after stroke onset. The reason could probably explain why we found a significant association between the increased level of serum S100B protein and initial NIH scores as an early neurologic outcome. If we take this finding into account, we could suggest that to determine the relations between long-term neurological survival rate and S100B protein serum levels, the blood sampling could be done 48-72 hours after stroke onset.

In pathophysiological analyses of various diseases has showed that TRAIL could paly role on inducing apoptosis of endothelial cells and inflammation of the arterial wall and promoting atherosclerotic plaques formation ^20^.

Recently, researchers have shown that serum TRAIL (sTRAIL) levels correlated with stroke outcomes that evaluated at first week after stroke onset ^21^. Similarly, another study found that low sTRAIL levels was associated with stroke severity in 293 patients seven days after stroke onset ^22^. In a different way, here we showed the biological significance of sTRAIL inversely related to both early and late neurological prognosis, final infarct core, and the presence of large artery atherosclerosis within 24 hours of stroke onset suggesting that TRAIL might be involved in neuroprotectiveprocesses in ischemic stroke.

Adropin plays a crucial role in vascular health and insulin sensitivity ^16^. Wu, *et al*. discovered that decreased adropin levels in diabetic patients were significantly correlated with coronary atherosclerotic damage as compared with non-diabetic ones among 392 patients with acute coronary syndrome ^23^. In an experimental study, the researchers have suggested that the increased expression of adropin might positively affect plaque stability and vascular elasticity to attenuate atherosclerosis ^24^. Gu, *et al*. discovered that adropin was negatively correlated with primary hypertension in 123 newly diagnosed patients ^25^. Similarly, the researchers reported that patients with cardiac syndrome X had lower adropin levels than other study patients ^26^. Altamimi, *et al*. concluded that adropin had a positive effect on cardiac metabolism associated with insulin resistance in an experimental study ^27^. In our study, we found that low adropin levels were associated with a large infarct core and initial and long-term disability and that all stroke patients with diabetes had decreased adropin levels.

Atherosclerosis is an important mechanism in LAA stroke and the process involves the immune system response and vascular inflammation ^14^. Secchiro, *et al*. showed that plasma TRAIL levels were lower in patients with myocardial infarction ^28^, and Cartland, *et al*. concluded that TRAIL protected against atherosclerosis by reducing inflammatory cells ^29^. Similarly, Sato, *et al*. suggested that adropin could contribute to antiatherosclerosis by modulating inflammatory molecule expression and smooth cell proliferation ^24^. Indeed, clinical studies have found that adropin plasma levels could contribute to the prevention of coronary artery diseases ^30^. In our three- month follow-up, we found that TRAIL and adropin plasma levels negatively correlated with LAA prognosis as measured by mRS. Therefore, we suggest that TRAIL and adropin may be involved in the LAA stroke prognosis and that plasma levels of the biomarkers under study may play a significant role in predicting LAA stroke patients’ prognosis.

Our study had several limitations. First, the sample size was small and the predictive cut-off values of serum adropin and TRAIL need to be further confirmed in large-cohort studies. Secondly, studies using other techniques, such as carotid ultrasonography to measure the intima-media thickness of the common carotid artery, could be included to evaluate the relationship between subclinical atherosclerosis and the serum biomarkers and to determine high-risk patients without any clinical manifestations. Thirdly, further studies should evaluate more specially the level of these biomarkers in the samples of atherosclerotic plaque, carotid tissue, or saliva rather than serum levels. Providing saliva as a sample for adropin and TRAIL measurements would be advantageous as it would allow for non-invasive sampling compared to invasive blood sampling for serum levels testing. Finally, our study results could only showed single-timing serum adropin-TRAIL levels our measurements in acute stroke patients, so further studies should be designed to determine temporal changes in these biomarker levels.

Here we determined the changes of S100B, TRAIL, and adropin levels on atherothrombotic cerebrovascular disease. S100B, TRAIL, and adropin levels correlated inversely in acute stroke patients. Large infarct core and unfavorable early and late neurologic outcomes were significantly associated with the study biomarkers, which taken as the sole biomarker are not sufficientfor which it is necessary to combine them with clinical risk scores and neuroimaging data in stroke patients. Further could evaluate that Adropin and TRAIL might be a potent therapeutic agent in acute stroke patients due to potential roles in cellular signaling pathways that lead to pathogenesis and/or treatment of stroke.
